# Protective Effects of Morin Hydrate on Acute Stress-Induced Behavioral and Biochemical Alterations in Mice

**DOI:** 10.29252/NIRP.BCN.9.3.195

**Published:** 2018

**Authors:** Elizabeth Toyin Olonode, Adegbuyi Oladele Aderibigbe, Olusegun Adebayo Adeoluwa, Abayomi Mayowa Ajayi

**Affiliations:** 1. Department of Pharmacology and Therapeutics, College of Medicine and Health Sciences, Afe Babalola University, Ado Ekiti, Nigeria.; 2. Department of Pharmacology and Therapeutics, College of Medicine, University of Ibadan, Ibadan, Nigeria.

**Keywords:** Morin hydrate, Stress, Restraint, Anxiety, Depression

## Abstract

**Introduction:**

As stress affects the brain both physiologically and chemically, researchers try to find novel anti-stress compounds with beneficial therapeutic effects. In this regard, the effect of stress and its modulation by Morin hydrate was studied using different acute models in mice.

**Methods:**

The models employed were anoxic tolerance, swimming endurance, and acute restraint test. Morin hydrate or the vehicle was administered 30 minutes prior to each stress exposure while in the acute restraint test; the animals were pretreated for 7 days with Morin hydrate, vehicle, imipramine, or diazepam before stress exposure. The measured parameters were the onset of convulsion and immobility time in the anoxic tolerance and swimming endurance test, respectively, while in the acute restraint test, the animals were assessed for stress-induced anxiety using the elevated plus maze and depression using the forced swim test. Thereafter blood was withdrawn from the retro-orbital plexus and plasma separated, the brain was also isolated, homogenized, centrifuged, and the supernatant was obtained for biochemical estimation.

**Results:**

Morin hydrate (5, 10, 20 mg/kg) produced a significant reduction in immobility time in the swimming endurance test, while significantly increased the anoxic stress tolerance time. Acute restraint stress caused a significant decrease in reduced glutathione levels (which was reversed by Morin hydrate) and increased the level of malondialdehyde, a thiobarbituric acid reactive substance which is an index of oxidative stress and nitrite. These effects were attenuated by Morin hydrate. Also, pretreatment with Morin hydrate attenuates acute restraint stress-associated anxiety and depression, reversed the hyperglycemia evoked by the stressful exposure and normalized serum cholesterol levels.

**Conclusion:**

These findings suggest that Morin hydrate exhibits anti-stress effects and may be useful in the relief of stress.

## Highlights

- Stressful life occurrences contributes to the development of anxiety and depression.
- Acute restraint stress induced hyperglycemia and enhanced generation of free radicals.
- Morin hydrate reduces anxiety and depressive-like behaviours in mice.
- Morin hydrate normalized blood glucose, suppresses lipid peroxidation and oxidative stress in mice brain.

## Plain Language Summary

Stress is a feeling of physical, mental or emotional strain imposed by life’s situations and which affects the well-being of individuals. Hence the need to seek therapeutic means of relieving stress in order to maintain state of mental and physical well-being. The study seeks to assess the effect of morin hydrate on some detrimental effects of stress. Morin hydrate is a natural compound gotten from the plant Morus alba. It has also been found to be a constituent of red wine and some fruits such as guava, almonds and figs. To evaluate Morin hydrate protective and mitigating effects on stress, we employed some behavioural stress paradigms such as anoxic tolerance, swimming endurance and acute restraint tests that can replicate stressful life events in animals. We also evaluated some biomarkers of stress (Glutathione, malondialdehyde, Thiobarbituric Acid Reactive Substance, blood glucose level and serum cholesterol level) to further validate our claim of stress mitigating effect of morin hydrate. Having pre-treated animals with morin hydrate and subjecting them to different tests, we observed that stress associated behaviours such as anxiety, depression and fatigue were reversed and biochemical alterations triggered by stressful exposure were normalized all by morin hydrate treatment. These findings suggest that Morin hydrate exhibits anti-stress potentials and may be relevant for use in the relief of stress.

## 1. Introduction

Stress is a state or feeling of being overwhelmed by demands exceeding the personal and social means of coping in the prevailing situation (Doreddula et al., 2014). Living organisms faced with stressful situations undergo some physiological, morphological, and biochemical modifications in order to survive. These modifications are evolved to reduce the demands and maintain the homeostatic environment through a series of physiological and/or behavioral responses (Morgan & Tromborg, 2007; Kothiyal & Ratan, 2011), but insufficient or excessive and/or extensive activation of the stress system can perturb the innate physiological and behavioral function (Tilbrook & Clarke, 2006).

Proper and effective stress management starts with identifying the stressor, its symptoms and effect on one’s health. Poor stress management contributes to the development of various psychiatric ailments, including, schizophrenia, anxiety, depression, post-traumatic stress disorders, bipolar disorder, Alzheimer disease, Parkinson disease and pathologic aging (Heim & Nemeroff, 1999; Joshi, Sah, & Singh 2012; Marin, et al., 2011). A wide variety of drugs are currently used to reduce stress, but overdependence or misuse of these drugs are often associated with side effects that may limit their therapeutic usefulness. Adaptogens are herbal preparations or formulations which places an organism into a state of generalized elevated resistance to stress by promoting homeostasis (Lakshmi & Sudhakar, 2009). They are used in the indigenous system of medicine for the management of stress because they increase the body’s resistance to stress by normalizing the production of stress hormones and enhancing the body’s ability to cope with anxiety and fight fatigue without or with minimal alterations in physiological functions (Panossian, Wikman, & Wagner, 1999).

Flavonoids are distributed vastly in the plant kingdom and can be found as constituents of food products, beverages and herbal preparations and have been shown to possess various health benefits (Catarino, Alves Silva, Pereira, & Cardoso, 2015; Li, Liu, Zhang, & Yu, 2008). Biological activities are attributable to the naturally occurring flavonoids, as well as some of their synthetic derivatives include anti-stress (Tiwari, Mishra, Bhatt, & Chaudhary, 2015; Watanabe & Ayugase, 2008), anti-oxidant (Zhang, et al., 2009), anti-inflammatory (Galvez et al., 2001), and neuroprotective compounds (Campos-Esparza, Sánchez Gómez, & Matute, 2009).

Morin hydrate (3, 5, 7, 2′, 4′-pentahydroxyflavone) is a flavonoid originally isolated from the branches of Morus alba L (white mulberry). It is ubiquitously distributed in the Moraceae plants family (Manna, Aggarwal, Sethi, Aggarwal, & Ramesh, 2007). It is also found in fruits like almond, sweet chestnut, guava, and apple, and can also be found in red wine (Basile et al., 2000; Wijeratne et al., 2006; Lotito & Frei, 2006; Rattanachaikunsopon & Phumkhachorn, 2010). Morin hydrate possesses diverse pharmacological properties. These include antioxidant (Wu et al., 1993; 1995; Prahalathan, Kumar, & Raja, 2012; Merwid-Lad et al., 2012), anti-inflammatory (Galvez et al., 2001; Fang et al., 2003), neuroprotective (Gottlieb et al., 2006; Ibarretxe, Sánchez Gómez, Campos Esparza, Alberdi, & Matute, 2005; Zhang et al., 2010; Campos-Esparza et al., 2009), and anticancer activity through inhibition of NF-кB activation (Manna et al., 2007).

Stress is associated with the generation of free radicals, release of excess amount of inflammatory cytokines and the activation of several inflammatory genes, including NF- кB. In view of its antioxidant, anti-inflammatory, and NF-кB inhibitory properties, it is postulated that Morin hydrate will serve as a potent anti-stress agent.

## 2. Methods

### 2.1. Laboratory animals

Male Swiss mice (22–25 g) were used in the study. They were kept at 20°C–25°C with free access to water and food and under a 12:12 h light/dark cycle. The animals were obtained from the central animal house, University of Ibadan and housed in groups in plastic cages. All behavioral tests were carried out between 8:00 AM and 1:00 PM. Mice were used according to the NIH Guide for the Care and Use of Laboratory Animals and the experiments were performed after approval of the protocol by the Ethics Committee of the University of Ibadan (UI-ACUREC/App/2015/067). All efforts were made to minimize animal suffering and to reduce the number of experimental animals used.

### 2.2. Drugs and chemicals

Morin hydrate was purchased from Sigma-Aldrich (Sigma-Aldrich, USA), imipramine from Gracure pharmaceuticals (Gracure pharmaceuticals ltd, India), and diazepam from Hoffman-La Roche (Hoffman-La Roche, Switzerland). Doses were determined on the basis of pilot studies and available literature. All drugs were dissolved in distilled water.

### 2.3. Experimental procedures

#### 2.3.1. Swimming endurance test

Swimming endurance test was performed according to Kannur et al. (2006) method with some modifications. Mice were divided into four groups (n=5). Group 1 or the control group received 10 mL/kg of the vehicle (distilled water), and groups 2 to 4 received 5, 10, and 20 mg/kg Morin hydrate, respectively, intraperitoneally 30 minutes prior to the test. Each animal was then transferred to transparent tank (30×45×40 cm) containing water at room temperature and observed for swimming. The total duration of immobility was noted for 15 minutes. Each mouse was judged to be immobile when it quit struggling and remained floating motionless in the water, making only those movements necessary to keep its head above water (Subarnas et al., 1993).

#### 2.3.2. Anoxic tolerance test

The anoxic tolerance test was carried out according to Kannur et al. (2006) method. Conical flasks of 250 mL capacity which were made air tight using rubber cork before the start of the experiment were used. Animals in this test were also divided into four groups (n=5). Group 1 (control) received 10 mL/kg of the vehicle, groups 2 to 4 received 5, 10, and 20 mg/kg Morin hydrate, respectively intraperitoneally. Thirty minutes after treatment, each mouse was kept in the conical flask and the delay for the first convulsion was observed.

#### 2.3.3. Acute restraint test

Acute restraint stress model was carried out according to the method of Masood et al. (2003) with minor modifications. The animals were allotted into six groups (n=6). Group 1 (non-stressed) received 10 mL/kg vehicle, group 2 (control) also received the vehicle, groups 3 to 5 received 5, 10, and 20 mg/kg Morin hydrate respectively while group 6 (positive control) were further divided into two subgroups. Subgroup A received 1 mg/kg diazepam and subgroup B received 12 mg/kg, imipramine. All treatments were carried out intraperitoneally for 7 days with the test compound or vehicle or the reference drug and on the seventh day, 30 minutes after the last dose, the animals in groups 2 to 6 were exposed to stress by restraining them in a plastic restrainer (a 50 mL cylindrical tube with holes for air circulation) for 2 hours. At the end of the procedure, all animals were subjected to forced swim test and elevated plus maze test in order to assess for depression and anxiety-like behavior, respectively. Following behavioral test, the animals were anesthetized and blood was withdrawn from the retro orbital plexus for the estimation of total glucose, protein, cholesterol and triglyceride levels. The animals were then sacrificed through cervical dislocation and the brain was excised for the estimation of antioxidant parameters.

#### 2.3.4. The elevated plus maze

The elevated plus maze is a validated behavioral assay for assessing anxiolytic and anxiogenic-like activities of pharmacological agents in rodents (Walf & Frye, 2007). The apparatus is made of wood consisting of two open arms (30×5×0.25 cm) which are essentially unprotected boards and two closed arms (30×5×15 cm) which are bordered by walls emanating from a common central platform (5×5 cm) and elevated to a height of 50 cm above the floor level. Each mouse was placed onto the middle of the apparatus facing an open arm. The time spent in each arm and the number of entries into each arm was manually recorded by a blind observer for 5 minutes. At the end of each trial, the apparatus was wiped clean with 70% ethanol solution and dried in order to prevent olfactory cue. An entry was defined as when all four paws have crossed the line between the arm and the central area. Anxiolytic action was defined by increased time in and or number of entries to open arms (Walf & Frye, 2007).

#### 2.3.5. Forced swim test

Forced swim test was carried out according to Deepika et al. (2013) method with minor alterations. Briefly, mice were individually forced to swim in a transparent Plexiglas cylinder of dimension (37×37×30 cm) filled with water to a depth of 25 cm at 25°C. The total duration of immobility was noted during a 6 min test session by a blind observer. Each mouse is considered immobile when it ceased struggling and floats passively in water, only making movements necessary to keep its head above the water.

#### 2.3.6. Measurement of blood glucose

Blood glucose level was measured using a blood glucose monitoring meter (AccuCheck performer meter) and blood glucose strip (AccuCheck). The strip was placed into the meter and blood was dropped at the tip of it and drawn up into the meter. Immediately the blood glucose reading was displayed on the meter and recorded.

#### 2.3.7. Determination of serum total cholesterol

Serum cholesterol level was determined by enzymatic colorimetric method using the assay kit (Spinreact, Spain) according to the manufacturer’s protocol following the method earlier described (Young & Friedman, 2001). Briefly, the reagent (containing the enzymes cholesterol esterase, cholesterol oxidase, peroxidase, and 4-aminophenazone) was dissolved with the buffer to give the working reagent. The mixture was gently mixed to dissolve the contents. About 1.0 mL of the working reagent was mixed with 10 μL of serum or cholesterol standard (200 mg/dL) and the mixture was incubated for 10 minutes at room temperature. Afterwards, the absorbance (A) of the samples and standard is read against the blank at 500 nm. The concentration of cholesterol in the sample was obtained from the following equation:

$$\frac{(A)\;\mathrm{Sample}-(A)\;\mathrm{Blank}}{(A)\;\mathrm{Standard}-(A)\;\mathrm{Blank}}\times 200(\mathrm{Standard}\;\mathrm{Conc}.)=\mathrm{mg}/\mathrm{dL}\;\mathrm{Cholesterol}\;\mathrm{in}\;\mathrm{the}\;\mathrm{Sample}$$

#### 2.3.8. Determination of serum triglyceride levels

Serum triglyceride level was determined by enzymatic colorimetric method using the assay kit (Spinreact, Spain) according to the manufacturer’s protocol following the method earlier described (Bucolo & David, 1973). About 1 mL of the reagent is mixed with 10 μL of the sample or standard and the mixture incubated for 5 minutes at 37°C. Absorbance (A) is read at 500 nm. Concentration (mg/dL) of triglyceride in the sample is obtained from the equation:

(A) Sample/(A) Standard×200 (Standard Conc.)

#### 2.3.9. Protein estimation

Protein estimation was carried out according to the method of Gornall et al. (1949). Briefly, 0.1 mL of sample is added to 0.9 mL of distilled water in a test tube, 3 mL of biuret reagent was added and the mixture incubated at room temperature for 30 min and absorbance was read at 540 nm. The standard (1 mg/mL bovine serum albumin) was measured in the range of 0.01–0.1 mg/mL.

#### 2.3.10. Determination of glutathione (GSH) concentration

Brain GSH concentration was determined using the method of Moron et al. (1979). Briefly, 0.1 mL of brain supernatant was added to test tubes containing 0.9 mL sodium phosphate buffer (0.2 M, pH 8.0). About 1 mL of 20% TCA containing 1mM EDTA was added and the mixture was allowed to stand for 5 min, and afterwards centrifuged at 10000 rpm for 10 min. Around 0.25 mL of the supernatant obtained was added to fresh test tubes containing 0.75 mL of phosphate buffer, and 2 mL of 0.6 mM DTNB was added. The mixture was allowed to stand for 5–10 min and absorbance was read at 412 nm. A standard curve of reduced glutathione was used to calculate GSH levels, which were expressed as μmol/g tissue.

#### 2.3.11. Determination of malondialdehyde in brain tissues

Determination of malondialdehyde (MDA), a biomarker of lipid peroxidation in brain tissue homogenate was carried out according to the method earlier described (Adam-Vizi and Seregi, (1982) in which MDA, an end-product of lipid peroxidation, reacts with Thiobarbituric Acid (TBA) to form a colored complex. Briefly, an aliquot of 0.1 mL of brain supernatant was added to plain tubes containing 1.9 mL of Tris–KCl buffer. About 0.5 mL of 30% TCA and 0.5 mL of 0.75% TBA were added to the mixture and incubated in a water bath at 96°C for 60 min. The mixture was then cooled under running water, then 2 mL of butanol was added and centrifuged at 3000 rpm for 10 min. After centrifugation, the reaction product was determined at 532 nm using MDA as standard. MDA values were expressed as μmol/g tissue.

#### 2.3.12. Estimation of brain nitrite level

Brain nitrite concentration was estimated using Greiss reagent, which serves as an indicator of nitric oxide production. About 300 μL of brain supernatant was added to test tube containing 100 μL Greiss reagent (1:1 solution of 1% sulfanilamide in 5% phosphoric acid and 0.1% of N-(1-Naphthyl) ethylenediamine dihydrochloride. Then, 2.6 mL of distilled water was added to the mixture and incubated at room temperature for 30 min. The blank was also prepared by adding 2.9 mL of distilled water to 0.1 mL Griess reagent and absorbance was read at 548 nm (Green et al., 1982). The brain nitrite concentration was estimated from a standard curve obtained from sodium nitrite (0–100 uM).

### 2.4. Statistical analysis

The results are expressed as Mean±S.E.M. The statistical significance was determined by 1-way ANOVA followed by Tukey post hoc test. Graph Pad Prism software version 4.03 was used for statistical analysis. P value of less than 0.05 was considered to be statistically significant.

## 3. Results

### 3.1. Effect of morin hydrate on immobility time in the swimming endurance test

**Figure 1:**
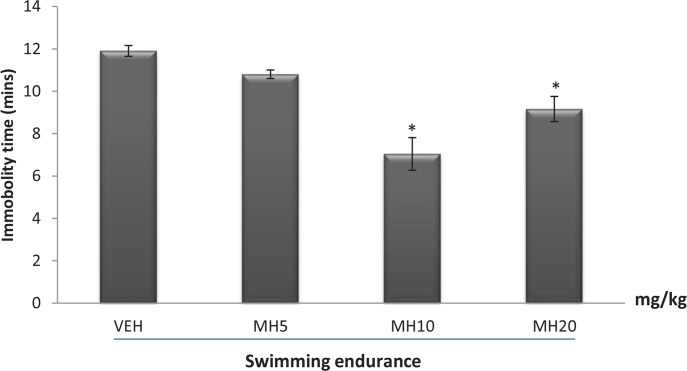
Effect of morin hydrate on the immobility time in the swimming endurance test Each bar is expressed as Mean ± SEM, (n=5 per group). * P<0.01 When compared to control group (ANOVA followed by Tukey post-hoc test); VEH: Vehicle; MH: Morin Hydrate

Figure 1 presents the effect of Morin hydrate on immobility time in the swimming endurance test. One-way ANOVA revealed a significant difference among treatment groups (F_3,16_=17.10, P<0.0001). Post hoc comparisons indicated that doses 10 and 20 mg/kg increase swimming endurance by reducing immobility time compared to VEH group (Tukey post hoc comparisons, P<0.01).

### 3.2. Effect of morin hydrate on anoxic stress tolerance

**Figure 2:**
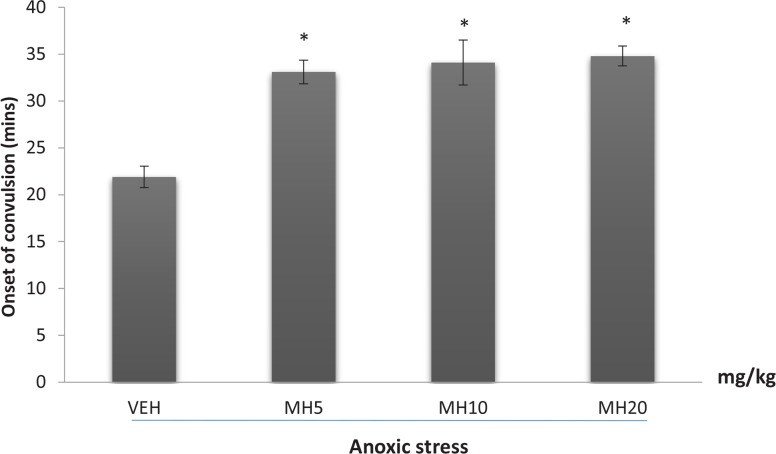
Effect of morin hydrate on anoxic stress tolerance Each bar represents the mean±SEM, (n=5 per group); * P<0.001 When compared to control group (ANOVA followed by Tukey post-hoc test); VEH: Vehicle; MH: Morin Hydrate

The effect of Morin hydrate on the onset of convulsion in mice subjected to anoxia is shown in Figure 2. Oneway ANOVA revealed a significant difference among treatment groups (F_3,16_=15.20, P<0.0001). Post hoc comparisons indicated that all doses were effective in increasing anoxic tolerance compared to VEH (Tukey post hoc comparisons, P<0.001).

### 3.3. Effect of morin hydrate on the duration of immobility in the forced swim test

**Figure 3:**
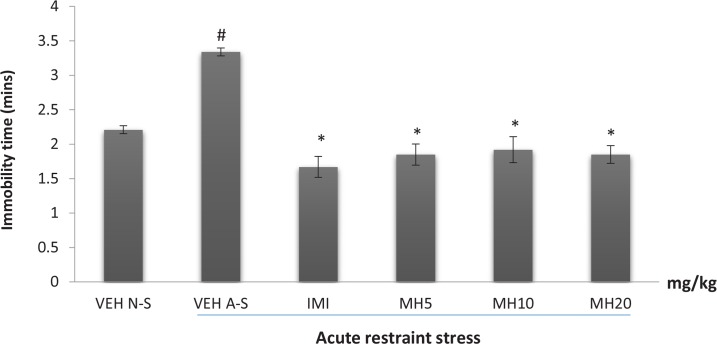
Effect of morin hydrate on the duration of immobility in the FST after acute restraint stress Each bar represents the Mean ± S.E.M; # P<0.001 When compared to control group; * P<0.001 When compared to the untreated group (ANOVA followed by Tukey post-hoc test); VEH N-S: Vehicle Non-Stress; VEH A-S: Vehicle Acute Stress; IMI: Imipramine; MH: Morin Hydrate

The effect of Morin hydrate on stress-induced depressive-like behavior as measured by the duration of immobility in the forced swim test is shown in Figure 3. One-way ANOVA revealed a significant difference in immobility time among treatment groups (F_5,12_=6.952; P<0.0029). Acute restraint stress caused a significant increase in the duration of immobility in the VEH acute stress group when compared to VEH non-stress group (Tukey post hoc comparison, P<0.001) (Figure 3). The reference drug, imipramine significantly reversed immobility as did all the doses of MH (each group compared to the vehicle acute stress group) (Tukey post hoc comparison, P<0.001).

### 3.4. Effect of morin hydrate on the elevated plus maze

**Figure 4:**
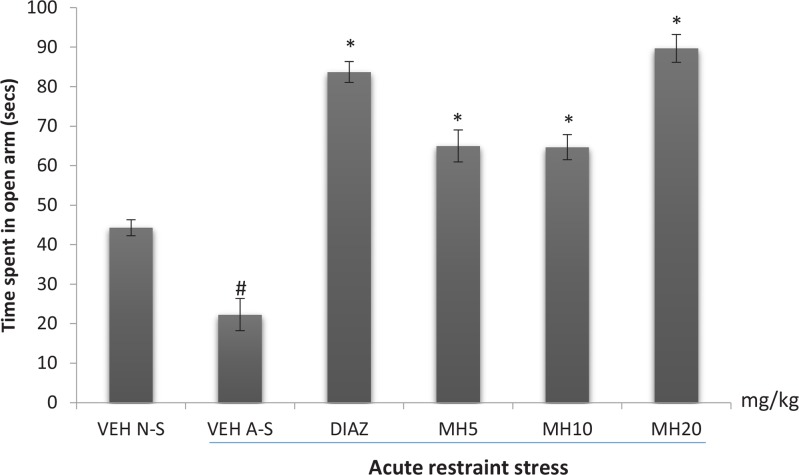
Effect of morin hydrate on time spent in the open arm of the EPM after acute restraint stress Each bar is expressed as Mean±S.E.M; # P<0.001 When compared with the control; * P<0.001 When compared with the stressed untreated group (one-way ANOVA followed by Tukey post-hoc test); VEH N-S: Vehicle Non-Stress; VEH A-S: Vehicle Acute Stress; DIAZ: Diazepam; MH: Morin Hydrate

The effect of Morin hydrate on stress-induced anxiety-like behavior as measured by the time spent in the open arms is shown in Figure 4. One-way ANOVA revealed a significant difference among treatment groups (F_5,12_=69.51; P<0.0001). The VEH acute stress group spent significantly less time in the open arm compared to the VEH non-stress group. Diazepam significantly reversed this effect as well as Morin hydrate at all doses, and even made the mice less anxious than the vehicle non- stress mice (Tukey post hoc comparison, P<0.001).

### 3.5. Effect of morin hydrate on acute restraint stress-induced changes in biochemical parameters

**Figure 5:**
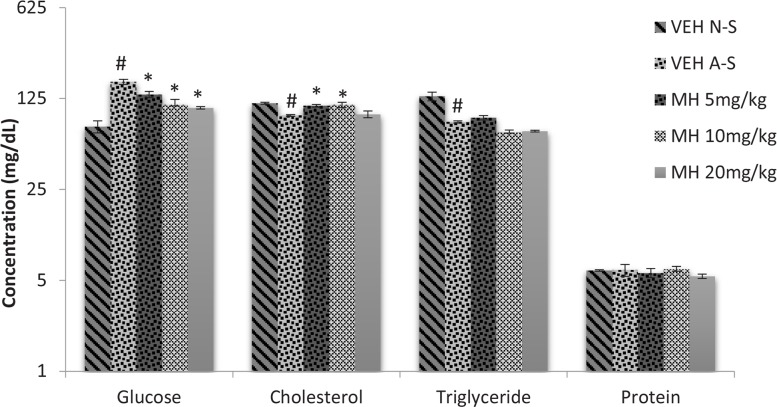
Effect of morin hydrate on acute restraint stress-induced changes in biochemical parameters Each bar is expressed as Mean±SEM; # P<0.05 When compared to control group; *P<0.05 When compared to the stressed untreated group (ANOVA followed by Tukey post-hoc test); VEH N-S: Vehicle Non-Stress; VEH A-S: Vehicle Acute Stress; MH: Morin Hydrate

Acute restraint stress raises serum glucose level (Figure 5). One-way ANOVA revealed an overall difference among treatment groups (F_5,12_=12.71, P<0.0001). A significant increase in serum glucose level in VEH acute stress group was observed compared to non-stress group (Tukey post hoc comparisons, P<0.05). Mori hydrate at all doses significantly (P<0.05) reversed this effect. Furthermore, 1-way ANOVA indicated that acute restraint stress significantly decreased cholesterol (F_4,10_=7.433, P=0.0048) and triglyceride (F_4,10_=24.64, P<0.0001) levels compared to VEH non-stress group. Morin hydrate (at doses 5 and 10 mg/kg) significantly reversed this effect on cholesterol levels, while no significant effect was observed on triglyceride and protein levels (Tukey post hoc comparisons, P<0.05).

### 3.6. Effect of morin hydrate on oxidative measurements

**Figure 6:**
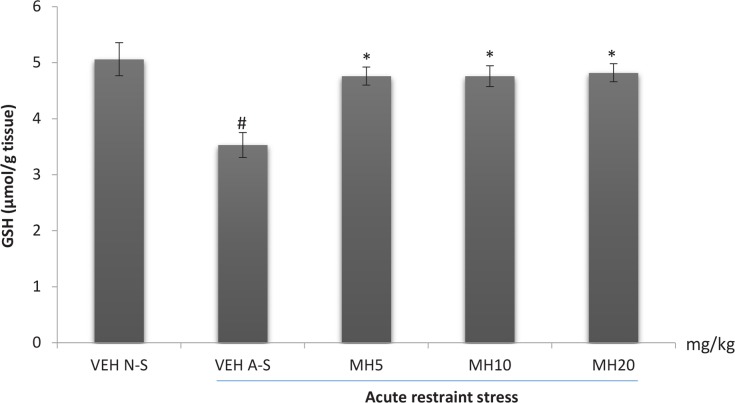
Effect of Morin hydrate on GSH levels in mice brain exposed to acute restraint stress Each bar is expressed as Mean±S.E.M; # P<0.01 When compared with the control; * P<0.05 When compared with the untreated group (one-way ANOVA followed by Tukey post-hoc test); VEH N-S: Vehicle Non-Stress; VEH A-S: Vehicle Acute Stress; MH: Morin Hydrate

**Figure 7:**
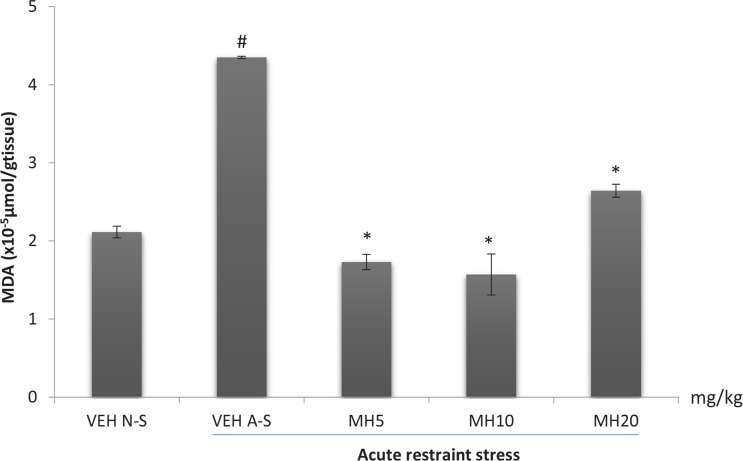
Effect of morin hydrate on MDA levels in mice brain exposed to acute restraint stress Each bar is expressed as Mean±S.E.M; # P<0.001 When compared with the control; * P<0.001 When compared with the stressed control group (one-way ANOVA followed by Tukey post-hoc test); VEH N-S: Vehicle Non-Stress; VEH A-S: Vehicle Acute Stress; MH: Morin Hydrate

**Figure 8:**
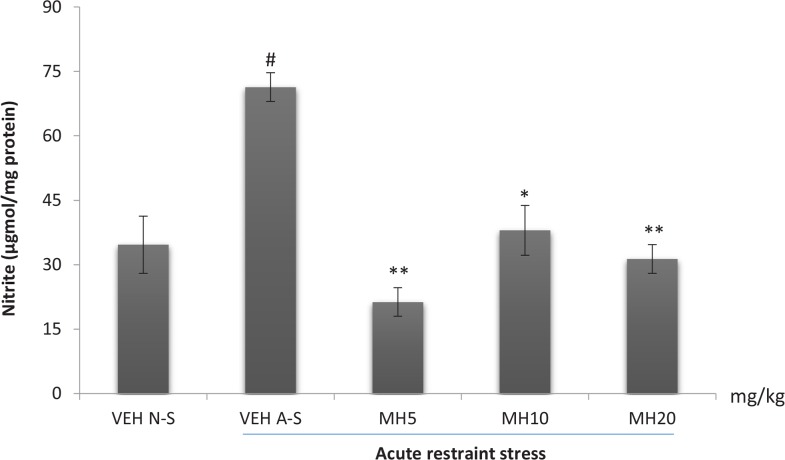
Effect of morin hydrate on nitrite levels in mice brain exposed to acute restraint stress Each bar is expressed as Mean±S.E.M; # P<0.01 when compared with the control; *P<0.01; ** P<0.001 when compared with the stressed control group (one-way ANOVA followed by Tukey post-hoc test); VEH N-S: Vehicle Non-Stress; VEH A-S: Vehicle Acute Stress; MH: Morin Hydrate

One-way ANOVA revealed a significant difference in the levels of brain GSH (F_4,10_=8.166, P=0.0034), MDA (F_4,10_, P<0.0001), and nitrite (F_4,10_, P=0.0002) among treatment groups. Acute restraint stress produced a significant reduction in brain GSH levels in the VEH acute stress group compared to the VEH non-stress group as shown in Figure 6. Morin hydrate at all doses significantly reversed the reduction in GSH levels (Tukey post hoc comparison, P<0.05). Furthermore, acute restraint stress caused a significant increase in brain MDA and nitrite levels in the VEH acute stress group when compared to VEH non-stress group (Tukey post hoc comparison, P<0.001 and P<0.01 respectively) as shown in Figures 7 and 8, respectively. All doses of Morin hydrate significantly reversed this effect (each group compared to the VEH acute stress group).

## 4. Discussion

The physiological changes induced by stress under normal circumstances are usually self-limiting and adaptive, but when the stressful event overrides its ‘threshold’ limits, it becomes irreversible, resulting in the pathogenesis of several disorders. The basic trigger for these disorders is the exhaustion of energy supply and breakdown of energy metabolism following circulatory glucose deprivation (Singh & Yadav, 2014). In the desire to enhance the coping mechanism, the science of adaptation focuses on expounding mechanisms that may help counteract exaggerated and unnecessary responses to stress (Rai et al., 2003). These adaptogens normalize and strengthen body systems and functions that have been compromised by stress, and provide protective effects against a wide variety of stressors. In general, they enhance the organism’s ability to adapt and evade damages incurred from stressful exposure (Panossian et al., 1999).

The swimming endurance paradigm is the most widely used model for evaluating the adaptogenic property of a novel compound (Anisman & Zacharko, 1991; Subarnas et al., 1993). This model is based on the observation that rodents, when forced to swim in a restricted space, become immobile after an initial period of vigorous activity signifying stress (Umukoro and Aladeokin, 2010). In the swimming endurance model conducted, a significant reduction in immobility time was observed at all doses administered. This increase endurance could be due to normalization of blood catecholamine and monoamine oxidase levels, a decline in muscle glycogenolysis, or a decrease in muscle concentrations of two toxic by-products of muscular efforts; lactic acid and ammonia (Debnath et al., 2011). It may also be attributed to increase in the utilization of the Adenosine Triphosphate (ATP) pathway or the anti-oxidant effect of Morin hydrate which prevents free radical-induced damage to vital organs.

Oxygen is a vital element on which all body functions including cellular respiration are dependent. Anoxic stress tolerance is characterized by shortage of oxygen supply which depicts an environmental stressor and any drug which increases adaptation under anoxic condition by increasing tolerance can act as an adaptogenic agent (Singh & Yadav, 2014). The significant increase in anoxic tolerance time could imply either resilience to anoxia or reduction in cerebral oxygen consumption, effects that are very beneficial in protecting neuronal cells against oxidative damage. In this study, Morin hydrate produced a dose-dependent increase in anoxic stress tolerance time, indicating its anti-stress activity. This observed prolongation of mean convulsion time could be caused by the antioxidant and free radical scavenging activity of Morin hydrate.

Restraint is one of the best explored models of stress in mice. This paradigm which restricts mobility and triggers aggression is a model of physical and emotional stress, and could trigger some psychiatric disorders such as anxiety and depression (Kulkarni & Juvekar, 2008). Anxiety and depression are the most prevalent psychiatric diagnosis in patients visiting psychiatric clinics (Gautam et al., 2012). Based on evidence, association between markers of stress and these psychological disorders has been amassed. The elevated plus maze is a validated model widely used for assessing anxiety-like behavior and elucidating mechanisms underlying anxiety-like behavior in rodents. The test is based on the natural avoidance of mice for open and elevated spaces, as well as on their natural spontaneous exploratory behavior in novel environments (Rodgers & Cole, 1993). The paradigm uses an elevated, plus-shaped apparatus with two open and two closed arms. Anxiety is expressed by a rodent spending more time in the enclosed arms compared to the open arms. Anxiolytic drugs have the characteristics of reducing anxiety reactions of rodents in elevated plus maze test. Animals treated with an anxiolytic tend to explore and spend more time in the open arm as compared with their untreated counterparts. The current study indicated that restraint stress caused a reduction in the time spent in the open arms which was reversed by treatment with Morin hydrate, suggesting that it could reduce fear and anxiety experienced after exposure to stressful conditions.

The FST model, like the swimming endurance test is based on fear or helplessness behavior in response to some inescapable and confined space and is sensitive to various antidepressant drugs. The alteration observed in the acute stress-induced immobility in the FST suggests that Morin hydrate attenuates stress-induced depressive-like behavior.

Increase in glucose concentrations in mice subjected to restraint stress has been previously reported (Sugimoto et al., 1998), and the result of this study agrees with the report. Stress-induced activation of the sympathetic nervous system stimulates the cells of the adrenal medulla to release adrenaline. Adrenaline has been reported to possess hyperglycemic effect as a result of its ability to suppress insulin secretion from the β-cells of the pancreas, resulting in elevated blood glucose (Shimazu, 1981; Kioukia-Fougia, 2002). Furthermore, adrenaline could stimulate adenylyl cyclase in the adipose tissue and muscle, resulting in an elevation in intracellular level of cAMP, whose role is to facilitate the mobilization of glucose and fatty acid reserves in tissues. Increase in cAMP levels results in a net increase in hepatic glucose production by stimulation of phosphorylase activation, suppression of glycogen synthetase activity, and stimulation of gluconeogenesis (Sutherland & Rodriguez, 1989). The significant elevation of blood glucose may also be as result of restraint stress-induced secretion of corticosterone from the adrenal glands as corticosterone has been reported to induce hyperglycemia and cause insulin resistance independent of glucose transport (Nicolson, 1997; Wass et al., 1996; Tappy et al., 1994). In this study, Morin hydrate was found to reverse the hyperglycemic effect observed after acute restraint.

Changes in the levels of particular lipids in response to stress have been recognized but the patterns and types of stress causing these changes have not been consistent. Stress-induced corticosterone release reduces the levels of triglycerides and cholesterol. This is due to the conversion of stored glycogen into glucose and utilization of fat reserves as alternative source of energy during stressful conditions (Kioukia Fougia, 2002). This reduction in serum triglyceride could also be as a result of the action of hormone-sensitive lipase which is triggered by adrenaline to mobilize fatty acids from adipose tissue triglycerides. In this study, restraint stress reduced serum cholesterol and triglyceride levels, while treatment with Morin hydrate normalized the cholesterol level but with no effect on triglycerides. This is probably due to diversion of energy substrates to the specific stress demanding sites (Singh et al., 2001).

Stress has been well documented to enhance the production of free radicals (Liu et al., 1994). Increase free radicals has been linked to hyper-activation of the hypothalamic-pituitary-adrenal axis with resultant increase in corticosterone secretion (Liu et al., 1996). Several reports suggested the critical role of stress-induced free radicals in several biochemical imbalances and their associated pathological outcomes (Olivenza, 2000). The analysis of lipid peroxidation revealed the occurrence of oxidative damage since an increased level of MDA was observed in the brain of stressed mice. The brain is largely sensitive to peroxidase damage due to its high oxygen tension, low antioxidant capacity and rich supply of oxidizable substrates (Metodiewa & Koska, 1999). Lipid peroxidation enhances depletion of intracellular endogenous antioxidant; glutathione, which serves as a first line of defense against oxidative assault. Morin hydrate at all administered doses reduced lipid peroxidation, lowered nitric oxide levels, and reversed the restraint stress-induced depletion in brain glutathione content. This further demonstrates the stress-relieving potential of Morin hydrate.

In conclusion, Morin hydrate increased resistance in the swimming endurance test and protects against anoxic stress. Furthermore, Morin hydrate protects against stress induced anxiety and depressive-like behavior, reduces hyperglycemia, and normalizes blood lipid levels. Finally, it protects against oxidative stress.

## Ethical Considerations

### Compliance with ethical guideline

Mice were used according to the NIH Guide for the Care and Use of Laboratory Animals and the experiments were performed after approval of the protocol by the Ethics Committee of the University of Ibadan (UI-ACUREC/App/2015/067). All efforts were made to minimize animal suffering and to reduce the number of experimental animals used.
